# Notes and News

**Published:** 1888-09-29

**Authors:** 


### NOTES AND NEWS.

COLLECTED AND COMMUNICATED.

IT is stated that of every £1 expended by the Cork Street Hospital, Dublin, 14*s.* is spent on officials, and only 6*s.* on patients.

THERE is on the various canals and waterways in this country, a population of between 60,000 and 70,000. A short account of these inland navigators will be found on another page.

UNDER the patronage of Lady Bayley, a bazaar was held in the grounds of Hatherop Castle on behalf of the Fairford Cottage Hospital. The net proceeds to be handed to the treasurer amounted to about £200.

ACCORDING to the recent report of the Lunacy Commissioners, out of 82,643 lunatics, idiots and other persons of unsound mind, no less than 13·4 per cent., or more than 6,000 of the cases, may be traced to habits of intemperance.

HER Royal Highness the Princess Frederica has consented to give the prizes at a doll show which is to be held in the Central Hall of the Sick Children's Hospital, Great Ormond Street, on November 5th.

A MOVEMENT is on foot to perpetuate the memory of Dr. Luke Armstrong, formerly a physician of the Newcastle Infirmary. A substantial amount has already been collected or promised, and it is intended to give all classes of the community an opportunity of contributing. The memorial will probably take the form of a lectureship or scholarship of Comparative Pathology.

WE are desired by the secretary of The Hospitals Association, in reply to numerous correspondents, to state that Mr. E. T. Clifford's popular paper on the "Position and Prospects of the National Pension Fund for Nurses," will be read before the Association at St. Thomas' Hospital, on Wednesday, October 10th, at eight o'clock p.m. As the number of seats required promises to be very large, it is desired that all who wish to avoid disappointment will make early application to the secretary, at Norfolk House.

THIS age is nothing if not analytical. 10,571 separate coins were taken at the recent open-air concert held on behalf of the Wakefield Hospital. Of these 1 was a sovereign, 4 were half-sovereigns, 13 half-crowns, 20 florins, 153 shillings, 605 sixpences, 706 threepenny pieces, 6,764 pennies, 2,224 half-pennies, and one a postal order for a sovereign—if the last may be called a "coin." The estimated number of persons present was 15,000. Some of them evidently were there with the object of receiving charity, in the form of a concert for nothing. Between four and five thousand, if not more, must have shirked the collection boxes. Yorkshire clearly is not without its mean people.

AT Swansea, the Eye Hospital, which has hitherto been a separate institution, has been amalgamated with the General Hospital. This is a significant indication of what may be expected in other quarters. As the knowledge of the public in hospital affairs increases, it will be found impossible to support half a dozen hospitals where one would serve the purpose. The days of special hospitals, with rare exceptions, are undoubtedly numbered.

A GIRL named Mary Griffin, 13 years of age, was arrested lasted week at Plymouth, and charged on her own confession with having caused the death of a boy named Alfred Delafield, the son of a Plymouth tradesman, in June last. Griffin stated that she had decoyed the boy away from home to the citadel, and there pushed him through an embrasure, from which he fell to the ground, a distance of 30 feet. When found, the child had been almost entirely stripped of his clothing.

AT the recent Homœopathic Congress, Dr. Dyce Brown said "the tactics of the old school just now are to absorb the results of homœopathic teaching and to repudiate the source from which they are derived." Is not this to admit that the so-called "old school" is willing to employ any methods it may consider useful, whatever their source? and does not the "old school" thereby prove itself to be catholic in practice, and not sectarian? Why do not the Homœopaths follow such an excellent example?

SIR JAMES PAGET last week opened a new hospital at Great Yarmouth. The building, which has cost £10,000, has been constructed on the pavilion principle, and has accommodation for 44 in-patients. The institution was inaugurated by Sir James formally unlocking the great door with a silver-gilt key, specially prepared for the purpose. Among those present were the Mayor and Town Council, the Earl of Oxford, Sir E. Birkbeck, M.P., several friendly societies, and a considerable gathering of the general public.

THE Director of Public Health in Italy, Dr. Pagliano, has sent a circular to the Prefects of the various provinces throughout the kingdom setting forth the desirability of giving some elementary instruction in obstetrics to those who practise midwifery in rural districts without qualification of any kind. With this object it is proposed that practical courses shall be held, each lasting fifteen days, in the obstetric clinics of the universities and schools of midwifery. The instruction is to be of a strictly elementary character.

ADDITIONAL accommodation for pupil nurses is being prepared at the Manchester Workhouse Infirmary, Crumpsall. At this large infirmary, with an annual admission of between eight and nine thousand patients, nurses have been successfully trained for many years. Each pupil nurse pays a fee of £20, and enters into an engagement to serve one year in the infirmary, after which period, other nursing work with a good salary is provided for her. Applications should be addressed to the Lady Superintendent.

CHILD marriages in India are receiving attention in high quarters, with a view to their discontinuance. A case occurred at Sunderland last week which painfully illustrates the misery and depravation of character which often follow such unions. At the police-court of that town a girl named Uide was charged with drunkenness. When placed in the dock she wept bitterly, and, in broken English, endeavoured to explain that she was a native of Bombay, and had been married at the age of thirteen to a soldier, who had brought her to England and died, leaving her friendless and destitute.

THE following vacancies are announced this week, the amount of salary in each case being given after the name of the institution :—

**Resident Medical Officers.**

Borough Hospital, Birkenhead. Junior House Surgeon —£50 per annum, with board, and fees.
East London Hospital for Children, Shadwell, E. Resident Clinical Assistant.—Board ; no salary.
Great Northern Central Hospital, Holloway Road, N. House Physician.—£50 per annum, with board, etc.
Manchester Royal Infirmary (Monsell Fever Hospital). Assistant Medical Officer.—£100 per annum, with board.
Salop Infirmary, Shrewsbury. Assistant House Surgeon.—Board ; no salary.
St. Mary's Hospital, Paddington, W. Medical Superintendent. —£150 per annum, with board.
Stourbridge Dispensary. House Surgeon and Secretary.—£130 per annum.
The County Asylum, Dorchester. Assistant Medical Officer.— —£120 per annum, with board, &c.

**Non-resident Medical Officers.**

Central London Throat and Ear Hospital, Gray's Inn Road. Three Clinical Assistants.
City of Liverpool Hospitals, at Park Hill and Grafton Street. Visiting Physician.—£100 per annum.
Dental Hospital of London, Leicester Square. Lecturer on Dental Surgery and Pathology.
Great Northern Central Hospital, Holloway Road, N. Physician.
Westminster Hospital, S.W. Fourth Assistant Physician.

---

A BUDDHIST journal has been started in Japan. Its object is the propagation of Buddhism in the West. The Editor, in an article entitled "What We Mean," explains that Christianity is now rapidly declining both in Europe and America. Contact with Christians, it is stated, has not been favourable to Japanese morals. By the influence of the "so called Western civilisation" Japanese liquors, comparatively harmless, have been replaced by beer, wine, and whisky. The Editor asks, "What is the religion which is to fill the gap when the Christian faith has disappeared from the souls of the Western peoples?" In his judgment, the religious union of the Buddhist nations, and the introduction of their religious truths into the West, are the two most pressing affairs of the hour. Several European writers have expressed sympathy with the movement, and one of them is of opinion that the religion of Buddha is much needed in Europe.

---

On the subject of out-door relief for the working poor, "A District Visitor" sends the following letter to the *Morning Post*: "I have, with much interest, read this morning, in your paper, the report of the Poor-law Relief Committee, and having had some experience in a poor neighbourhood, I desire to make a few remarks with regard to the relief of able-bodied men by means of the labour yards. I give one instance from several similar ones in my district of a man who has a wife and four young children. He works during the summer in the fields at a weekly wage of 15*s*. His rent for two rooms is 2*s*. 3*d*. a week, leaving him 12*s*. 9*d*. to pay school fees and feed and clothe himself and family. He is a total abstainer, but can save nothing. His work comes to an end in October, and from then till March or April he has no work, with the exception of an odd day from time to time. During the last two winters he has regularly attended the labour yard at the Union, receiving 1*s*. 4*d*. a day, half in money and half in bread. Surely this is better than compelling him to break up his home, sell his furniture, and go with his wife and children into the Union, where, I imagine the cost of their maintenance would be, at least, equal to the out-door relief he would receive. It cannot be demoralizing for the man to work for the relief given to him, and it seems to me that it is degrading him to force him into the workhouse ; indeed, I believe he would let his wife and children starve first, and meanwhile he would be standing idle at the corner of the street, a prey to bitter thoughts and discontent, which are neither good for himself nor his country."

UP to the present time the workshop collections made on behalf of the Hospital Saturday Fund amount to about £7,000, being £300 in excess of the sums from the same source in former years. It is expected that collections will continue to come in until the end of next month.

---

SAID a nurse to a visitor at a children's hospital, "I think people would send us some of their children's left-off clothes if only they knew how much we wanted them ; and it does not matter if they are old—anything that will fit a child comes in useful. Old slippers would be especially useful. Care should be taken that nothing is sent about which there may be any possibility of infection."

---

THE publication of the Emperor Frederick's journal has been received as a great event at Vienna. The *Neue Freie Presse* says that if the late Emperor had written nothing but the words about his chief thought being for peace, he would have sufficiently proved of what value his life was to the German Empire. All the Vienna papers speak of him as a bright star which was prematurely extinguished.

---

COMMENTING on the proposal to photograph the eyes of the murdered woman, Annie Chapman, with the view of securing a portrait of the murderer, the *Photographic News* says : "Dr. G. B. Phillips, official police surgeon, called as a medical witness, was asked as to the possibility of obtaining a clue to the murderer by photographing the eyes of the dead woman ; but, as might have been expected, he gave no hopes of any useful result. Although we may pretty confidently say that photographs of the eyes of the murdered woman would have been useless, there can be no doubt whatever that a series of photographs of the body and of the mutilations ought to have been taken, and in the face of these it would have been far more difficult to conceal essential facts. It is to be hoped that even now the camera will be brought into requisition, especially as there are several experienced anatomists who are also competent photographers. Perhaps a carefully-made series of photographs, and the calling of such scientific experts as have distinguished themselves in the new art of abdominal surgery, might throw light on the extraordinary action of the authorities in neglecting the most obvious means of getting information or of recording facts, and also on the strange action of Dr. Phillips."

---

"THE prophetic dread," says a correspondent in the *Times*, "which Dr. Conolly had of the resumption, after his death, of the rough and coarse treatment of the insane by manacles would seem to be not groundless. . . He often feared that this might be so, inasmuch as it required high moral qualities to carry out his system in its entirety, whilst the 'restraint' practice ministered directly to the cupidity and self-indulgence of those who had care of the insane. . . Not only the 'black sheep,' but the far larger class of neutral tint, gladly avail themselves of a resource which renders the poor patient powerless, and saves the mental exertions required to soothe him by moral means. Moreover, a strait-waistcoat requires no food or wages, and is altogether a very cheap substitute for a kind and skilled attendant." These remarks contain the quintessence of the whole subject, and evidently condense the experience of an honest, capable, and sympathetic witness. "Moral suasion" with lunatics means time, mental exertion, and expense ; but it secures kindness for all and cure for many. "Strait-waistcoats" have no recommendations except those of saving the labour to the attendants, medical and otherwise, and expense to the establishment. They mean much cruelty to many helpless and most sad people, and very few cures.

